# Amputation of the Lower Jaw
*Glasgow Journal, No. V.


**Published:** 1829-04-01

**Authors:** 


XLVIII.

AMPUTATION OF THE LOWER JAW.*

Another of these bold operations has lately been performed by Dr. Anderson, of Glasgow, and the patient died. It is only within these twenty years that surgeons have ventured on the formidable step of amputating a great portion of the lower jaw, but during that period, Dupuytren, Lallemand, and Lisfranc, in France; Gräafe, in Germany; Drs. Mott, and M. Clelland, in America; Messrs. Cusack, Wardrop, Lizars, Liston, Syme, and lastly Dr. Anderson, in this country, have gathered laurels in this bloody field. It would not be an act of justice to the last named gentleman, who has removed more of the bone than any of his predecessors, to withhold his case in toto from our readers, but a sketch of the particulars is all that our limits will afford. The value of a case is in the inverse ratio to its scarcity or its curiosity, and the man who could give a specific for the tooth-ache, would confer a more valuable boon on the public, than he who had tied the aorta with success. But to the purpose.

The patient, a Mrs. Sutherland, 37 years of age, was admitted into the Glasgow Infirmary, on the 5th of May, 1823, for a fungus of the antrum of the left side, which began two years before, after a long-continued tooth-ache. Dr. Anderson destroyed the fungus by exposing the anterior wall of the antrum, removing the whole of it, scooping out the fungus partly with the finger, and partly with a lithotomy scoop, and burning the whole diseased surface with the actual cautery. A good part of the wound was healed by the first intention; but, owing to an attack of erysipelas, it required some time and country air to effect the complete cicatrization. During more than five years Dr. Anderson occasionally continued to visit this woman, during which time she suffered much from rheumatism, and broke the neck of the left femur, but had no return of the fungus in the antrum. In April last she complained of tooth-ache in the lower jaw, and a loose molar tooth was extracted, soon after which a fungus appeared in that situation. On the 5th of last September, Dr. A. saw the tumour, but did not feel warranted in operating on account of the state of her health, and the suspicion that the fracture of the femur had arisen from a malignant diathesis throughout the body. On the 23d of October, at the patient's request that something should be done, on account of the repeated hæmorrhages, Dr. A. advised a consultation with his colleagues of the Infirmary. At this time, a firm spongy tumour occupied the left side of the inferior maxillary bone, from the symphysis backwards to the angle. It felt soft and elastic—its upper surface was flat, sloughy, and indented by the teeth of the upper jaw—it pervaded the whole thickness of the bone, projecting below it towards the neck, where several small glands were felt enlarged, and above pressing inwards on the tongue, and outwards on the face—the grinders on this side were carious, and the whole of the incisor teeth loose—a thin fetid fluid constantly oozed from the mouth—and hæmorrhage had repeatedly occurred to such an extent as to induce syncope. The tumour was occasionally affected with gnawing pain which extended to the head, the countenance was sallow, the pulse 110 and small, the appetite bad, and the strength much reduced.

The consultation met, the result of which was, that a lodging was procured, and the operation performed on the 30th October, in the presence of Professor Burns, Drs. Young, Maclachlan, and Auchincloss, Messrs. Weir, Cowan, Candlish, &c. The steps of the operation we cannot express more concisely than the author himself, and therefore subjoin his own words.

"The ascending plate of the bone was felt to be sound beyond the tumour, and the operation was commenced with the intention of applying the saw a little above the angle on the left side, and at the canine tooth on the right side, by which it was expected that the whole of the diseased structure should be removed. Having seated her on a chair, I began by extracting the right canine tooth; but in doing this, the socket yielded so as to excite a fear that it was unsound, and I determined to include also the two anterior molares. The first incision ex-

* Glasgow Journal, No. V.

tended from the angle of the mouth to the lobe of the left ear. The knife was carried through the masseter muscle, which was speedily detached from the bone, and the whole substance of the cheek, being dissected from the anterior surface of the tumour, and from the chin, was turned downwards on the neck. A small straight saw, rounded off at the point, was now applied immediately posterior to the second grinder on the right side. When the bone had been about half divided in this way, it was snapped across with the forceps recommended by Mr. Liston. The same process was practised above the angle on the left side, and the bone, having the tumour attached to it, was then removed. Here I expected to have finished the operation; but on examining the anterior section of the bone, it was discovered, that notwithstanding the apparent soundness of its outer shell, its medullary cavity was filled with the same fungous growth with that which had protruded on the left side. A further exposure of the jaw, therefore, became necessary, and this was effected by detaching the cheek as far as the right angle, without any new external incision.

"It now became obvious, that although there was no fungous protrusion on this side, there was even more extensive disease than on the opposite, and the necessity for extirpating the whole was a duty equally unexpected and imperative.

"The previous removal of the bone on the left, permitted so much retraction of the cheek on the right side, that I at first attempted to complete the operation at the joint, from within the mouth; and in this, I believe, I could have succeeded, having no occasion here to apply the saw. But expedition became a most important object; and I therefore divided the cheek as on the left side, from the angle of the mouth as far *dextrad* as the masseter muscle. Having detached this muscle from the bone, I experienced some of the difficulty described by others, in separating the insertion of the temporal muscle from the coronoid process. In effecting this, and endeavouring to open the joint anteriorly, by depressing the divided end of the bone, it broke across at the neck, immediately below the articular process. I proceeded to divide the pterygoidæi, and other muscles, towards the pharynx and mouth, by carrying the knife forwards in close contact with the inner surface of the bone, until the whole was removed. Having accomplished this, and finding that the fracture below the condyloid process had proceeded from diseased softening, I laid hold of the small portion that remained with a pair of tooth forceps. Along these I carried the scalpel, with which the capsular ligament of the joint was opened, and the head of the bone extracted. The whole of the lower jaw was thus removed, except that portion of the ascending plate, with its processes, measuring an inch and a half, which remained above the application of the saw on the left side. This, on examination, was found to be the only sound part, and even here the nerve was afterwards discovered by the microscope to be more pulpy than usual. The bony structure was quite destroyed where the tumour had protruded on the left side. Throughout the whole of the right side, even to the joint, the bone was uniformly enlarged, and contained the same spongy substance as had been observed at the first section with the saw; no part, therefore, was unnecessarily removed.

"The hæmorrhage from this operation was less than I had anticipated, being more from the general vascular surface than from the division of important vessels. About a pound of blood was lost, and only two ligatures were found necessary. But the previous debility, and the urgent symptoms during the operation, were calculated to excite very serious alarm. She became excessively restless, alternately vomiting the blood which she had swallowed, and appearing about to suffocate from some obstruction about the larynx, probably similar to that which occurred to Professor Lallemand, viz. a reversion of the tongue from the contraction of the divided muscles, for which he was obliged to perform tracheotomy.

"The wound, which had a hideous aspect, was quickly closed, by hare-lip needles at the angles of the mouth, and stitches and plasters towards the ears. Dossils of lint were inserted on each side of the tongue, and the whole was supported from without by a compress and bandage. The pulse was feeble, but distinct; the dyspnœa subsided. She had fifty drops of laudanum in brandy and water, through an œsophagus tube; and was put to bed."

On the fifth day after the operation this large wound had completely united except at a single point, from which the discharge of pus did not exceed a drop at each dressing. On the evening of the 12th day she complained of pain at the upper part of the sternum, which returned several times dext day with a feeble pulse, cold skin, and dyspnœa. She gradually sank, and died at 8, p.m. on the 13th day after the performance of the operation.

On dissection, the union of the wound was found to be deep and complete, except at one spot, the size of a split pea, near the lobe of the left ear. The left antrum, from which the fungus had been formerly removed, was nearly three times its natural size, and contained about an ounce of yellow transparent fluid. It had no communication with the mouth—was lined with a firm, smooth, shining membrane—the anterior and upper walls were nearly an inch thick, but very soft; but towards the palate they were thin and almost cartilaginous. A fracture existed at the neck of the left femur, within the capsule, which had united by cartilage, without much shortening. In the upper part of the right side of the thorax there were found about eight ounces of sero-purulent effusion, whilst below these were firm adhesions between the lung, the pleura costalis, and the diaphragm.

Dr. Anderson subjoins some valuable remarks on the diseases of the antrum he has met with in practice, and the best means of performing operations for their cure. We are sorry that our limited space prevents our doing more than allude to them here. With respect to the operation for removal of the lower jaw, Dr. A. observes, that no uniform rule can be laid down on the mode of conducting the incisions, but recommends, for its simplicity and consequent facility, that adopted in the present instance, viz. laying open the mouth outwards from its angles.

Though we fully agree with Dr. A. that nothing is so much to be denounced in the character of a surgeon as a reckless desire for operating, yet we cannot but accord our tribute of praise to the author of the present case for the able and scientific manner in which the operation sppears to have been conducted. It is evident that the patient was recovering, and, in all probability, would have entirely recovered from its immediate effects, had not her life been cut short by one of those insidious inflammations of the chest, which so often step in and throw the sword into the balance between Death and the Doctor.

